# Suprachoroidal Delivery of Corticosteroid Slow-Release Implants for the Treatment of Cystoid Macular Edema

**DOI:** 10.1055/a-2541-2444

**Published:** 2025-06-16

**Authors:** Ben Asani, Jakob Siedlecki, Julian Klaas, Siegfried Georg Priglinger

**Affiliations:** Department of Ophthalmology, University Munich, LMU, Munich, Germany

## Introduction

While intravitreal corticosteroid slow-release implants (CSRIs) like dexamethasone (Ozurdex, Abbvie, North Chicago, Illinois, USA) and fluocinolone (Iluvien, Alpharetta, Georgia, USA) have revolutionized cystoid macular edema (CME) management 1 , 2 , anterior migration of implants poses risks of endothelial damage, necessitating posterior lamellar keratoplasty 3 . To date, there have been few treatment alternatives as effective or long-lasting as CSRIs for patients with a disrupted iris–lens diaphragm. Scleral fixation of CSRIs with sutures has become a good alternative, particularly for the fluocinolone implant, but this technique can be complex 4 . However, as the fluocinolone implant has a firm non-dissolving shell, scleral fixation is stable in the long term. Scleral fixation of the dexamethasone implant 5 , 6 is hardly feasible as the implant loses diameter during drug release, which makes the suture fixation around the implant too loose and dislocation of the implant likely. As cost coverage issues are less problematic with the dexamethasone implant than with the fluocinolone implant and the dexamethasone implant has stronger drug efficacy, an alternative form of application in eyes with a disrupted iris–lens diaphragm that does not require intravitreal delivery by scleral suturing would be desirable. In these cases, suprachoroidal placement of CSRI implants could provide a promising alternative, ensuring stable positioning and effective drug delivery.

## Case Description

A 60-year-old male patient with a history of chronic CME after previous pars-plana vitrectomy due to traumatic phakic lens dislocation was referred for treatment. The patient had a scleral flange fixated lens and large iridectomy as well as a history of a dexamethasone implant dislocation into the anterior chamber. The patient reported progressive visual decline despite previous standard therapies such as parabulbar injection of triamcinolone or an intravitreal suspension of dexamethasone, both of which had an effect for only a short duration. The preoperative examination revealed:

- central retinal thickness (CRT): 446 µm
- best-corrected visual acuity (BCVA): 0.6 logMAR
- intraocular pressure (IOP): 21 mmHg

The patientʼs condition rendered intravitreal corticosteroid implantation unsuitable due to the risk of repeat anterior chamber migration.

## Intervention

**Fig. 1:**
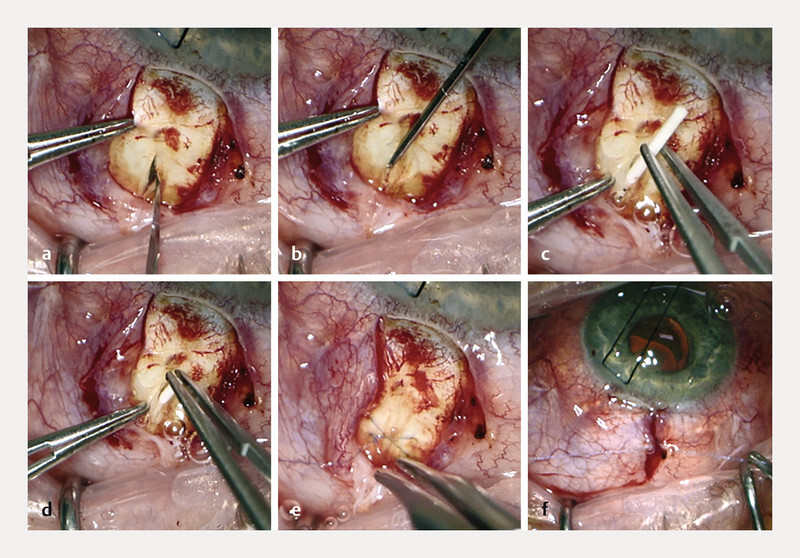
After conjunctival peritomy, a radial sclerotomy was created at the pars plana ( **a** ). Dispersive viscoelastic material was carefully injected into the suprachoroidal space ( **b** ). A sterile dexamethasone slow-release implant (Ozurdex, Abbvie, North Chicago, Illinois, USA) was released and placed into the suprachoroidal space ( **c** , **d** ). The sclerotomy and conjunctiva were sealed using a self-absorbing suture (8 – 0 vicryl, Ethicon Inc., Bridgewater, New Jersey, USA; **e** , **f** ).

The suprachoroidal implantation was performed under local anesthesia by an experienced surgeon (S. G. P.) under retrobulbar block as an outpatient procedure. After conjunctival peritomy, a radial sclerotomy at the pars plana was created ( Fig. 1 a ), followed by injection of dispersive viscoelastic material into the suprachoroidal space ( Fig. 1 b ). Then, a dexamethasone slow-release implant (Ozurdex, Abbvie, North Chicago, Illinois, USA) was released from the injector and then manually placed into the suprachoroidal space ( Fig. 1 c , **d** ). The sclerotomy and conjunctiva were sealed with a self-absorbing suture (8 – 0 vicryl, Ethicon Inc., Bridgewater, New Jersey, USA; Fig. 1 e , **f** ).

Postoperative imaging using swept-source optical coherence tomography (OCT) confirmed the correct placement of the implant. The procedure was completed without any intraoperative complications. The patient was instructed in postoperative care, including the use of topical antibiotics and steroids to prevent inflammation and infection.

## Outcome and follow-up

At the 1-month follow-up:

- CRT: reduced to 341 µm (ΔCRT = 105 µm)
- BCVA: improved to 0.2 logMAR
- IOP: stabilized at 19 mmHg

At the 6-month follow-up:

- CRT: 361 µm
- BCVA: stabilized at 0.2 logMAR
- IOP: 16 mmHg

The patient experienced complete resolution of macular edema, with substantial visual improvement. No adverse events or complications were observed during the follow-up period. The patient reported significant functional benefits, including improved reading ability, reduced visual strain, and greater independence in daily activities. The resolution of macular edema was sustained during the observation period at the 1-month and 6-month mark with only a slight increase in BCVA, without a need for retreatment, indicating the potential long-term efficacy of this approach.

Additionally, postoperative imaging using swept-source OCT (DRI OCT, Topcon Corporation, Tokyo, Japan) demonstrated stable implant positioning with no evidence of migration or degradation.

## Discussion

This case underscores the effectiveness of suprachoroidal delivery of dexamethasone CSRIs in managing CME in eyes with a complex anatomy. The suprachoroidal route minimizes the risk of anterior chamber migration while maintaining therapeutic efficacy. Postoperative imaging confirmed stable implant positioning, with no evidence of migration or adverse tissue reactions. The suprachoroidal space thus provides an optimal site for sustained drug delivery, allowing the corticosteroid to act directly on the choroid and retina with minimal intraocular absorption 7 .

Besides avoidance of anterior chamber complications, suprachoroidal dexamethasone delivery might offer the advantage of a reduced risk of IOP increase 8 . Additionally, risk of cataract formation might be substantially reduced in phakic eyes. Data from similar approaches with suprachoroidal triamcinolone (XIPERE, Rochester, NY, USA) have shown that anterior chamber steroid levels were much lower than expected if delivered to the intravitreal space 7 , 9 . In addition to eyes with an instable iris–lens diaphragm, the main adverse events from CSRIs like glaucoma and cataract could also be lessened for eyes susceptible to either complication (steroid-induced glaucoma or a clear lens in young patients). Additionally, this approach might reduce the risks associated with intravitreal injections, such as endophthalmitis or retinal detachment. However, it should be noted that endophthalmitis can also occur with suprachoroidal approaches and there are no treatment guidelines for this complication yet. The risk of suprachoroidal hemorrhage should also be mentioned, which can be more fulminant than the (rare) vitreous hemorrhage in intravitreal delivery.

While this single case demonstrated promising results, further research is needed to evaluate the long-term safety and efficacy of suprachoroidal corticosteroid implants. Randomized controlled trials with larger sample sizes could help establish this technique as a standard treatment option for CME in complex cases. Additionally, advancements in implant design and surgical techniques may further enhance the outcomes and accessibility of this approach. Incorporating patient-reported outcome measures in future studies could also provide valuable insights into the real-world benefits of this treatment.
